# A Novel Biological Dosimetry Method for Monitoring Occupational Radiation Exposure in Diagnostic and Therapeutic Wards: From Radiation Dosimetry to Biological Effects

**Published:** 2016-03-01

**Authors:** S. Heydarheydari, A. Haghparast, M.T. Eivazi

**Affiliations:** 1 Department of Medical Physics, Faculty of Medicine, Kermanshah University of Medical Sciences, Kermanshah, Iran; 2 Department of Medical Physics, Faculty of Medicine, Kermanshah University of Medical Sciences, Kermanshah, Iran; 3 Department of Medical Physics, Faculty of Medicine, Kermanshah University of Medical Sciences, Kermanshah, Iran

**Keywords:** Biological Dosimetry, Radiation Exposure, Radiation Dosimetry, Biological Effects

## Abstract

**Background and Objective:**

Professional radiation workers are occupationally exposed to long-term low levels of ionizing radiation. Occupational health hazards from radiation exposure, in a large occupational segment of the population, are of special concern. Biological dosimetry can be performed in addition to physical dosimetry with the aim of individual dose assessment and biological effects.

**Methods:**

In this biodosimetry study, some hematological parameters have been examined in 40 exposed and 40 control subjects who were matched by gender, age and occupational records (±3 years) in Kermanshah hospitals in Iran (2013-2014). The occupational radiation dose was measured by personal dosimetry device (film badges). The data was analyzed using SPSS V.20 and statistical tests such as two-sided Student’s t-test.

**Results:**

Exposed subjects had a median exposure of 0.68±1.58 mSv/year by film badge dosimetry. Radiation workers with at least a 10-year record showed lower values of Mean Hemoglobin (Hb) and Mean Corpuscular Volume (MCV) compared to the control group (p<0.05). The mean value of Red Blood Cells (RBCs) in personnel working in Radiology departments seemed to show decrease in comparison with other radiation workers.

**Conclusion:**

Although the radiation absorbed doses were below the permissible limits based on the ICRP, this study showed the role of low-level chronic exposure in decreasing Hb and MCV in the blood of radiation workers with at least 10 years records. Therefore, the findings from the present study suggest that monitoring of hematological parameters of radiation workers can be useful as biological dosimeter, and also the exposed medical personnel should carefully follow the radiation protection instructions and radiation exposure should be minimized as possible.

## Introduction

One of the significant changes in technology is employing electromagnetic waves in different parts of industrial science and medical appliances[1-3]. Ionizing radiation, particularly X-ray and those emitted by radioactive substances, play a vital role in medicine, both in diagnosing and treating diseases[4]. On the other side, ionizing radiation is known as one of the detrimental factors in the work environment that can cause serious, irreversible and irreparable damages in professional radiation workers, but the effects of low doses on human health has not been completely known[5-8,21].

Considering and following up the health of persons who are occupationally exposed to long-term, low levels of ionizing radiation are of great importance. Therefore, the information in case of a radiation accident comes especially from physical dose, blood count data (changes in hematological parameters) and from the clinical symptoms that exposed individuals might display[22].

The risk conception depends on the knowledge of potential health effects of ionizing radiation and the mechanisms employed to predict them. In this content, physical and biological dosimetries are the main tools for individual monitoring. Assessment of given biological endpoints to estimated or known absorbed dose can be used in retrospective studies of individual radiation exposure[23].

There was an increasing interest in biological parameters for low-level chronic radiation exposure observed among radiation workers[10, 11]. The radiation sensitivity of cells is different, and in fact hematopoietic cells are the most sensitive cells to radiation[9]. Therefore, complete blood cells counting can be used not only as an index to assess the extent of ionizing radiation damage on the hematopoietic system but also as a suitable biological indicator for investigating damages[12, 13].

Basic studies on the biological response to radiation at low doses are considered a research priority in order to better understand the occupational risks associated with working in radiation departments with the possible development of long-term health effects.

Considering and following up the health of persons who are occupationally exposed to long-term, low levels of ionizing radiation is of great importance. Nowadays, biological dosimetry is mainly performed based on the chromosome aberrations (CA) but this biological dosimetry method is time consuming and labor intensive. Therefore, the current study was performed to investigate the biological effects of occupational exposure based on hematological parameters. 

## Materials and Methods

The study group comprised totally of 80 samples which included 40 exposed subjects (26 females and 14 males) and 40 controls (26 females and 14 males). The exposed subjects were selected from radiotherapy, radiology and nuclear medicine departments in various hospitals of Kermanshah city of Iran (2013-2014) who were chronically exposed to low doses of ionizing radiation. Selected controls work in the same hospitals without being exposed to any kind of radiation doses. 

All exposed and control subjects match gender, age and occupational records (±3 years). They were categorized based on the duration of exposure as group 1 (<10 years) and group 2 (≥10 years). 

Their occupational exposure to ionizing radiation is routinely monitored by personal exposure measurement devices (film badges). The occupational record of the radiation worker group (case) was at least two years and also their exposure doses have not exceeded the maximum permissible dose (MPD) during the period of work time. 

Exclusion criteria for both exposed and control subjects included personal medical history, recent infectious status, use of some medicines such as acetaminophen or other painkillers and antibiotics at least one month before CBC test and finally smoking. 

CBC tests were performed on 2.5cc blood specimen of both groups (case & control). It was drawn from a vein located inside the elbow or the back of hand. First, the site was cleaned with antiseptic. Second, an elastic band is put around upper arm to apply pressure to the area in order to make the vein swell with blood. Third, a needle was inserted into the vein and the blood collected into an airtight tube attached to the needle. Then, blood samples were drawn into glass tubes containing ethylene diamineteraacetic acid (EDTA). Finally, blood samples were blended with EDTA by rotator. Samples were analyzed and counted by Sysmex cell counter machine (XT-1800i, Germany). The number of red blood cells, platelets, subpopulations of white blood cells as well as hemoglobin, hematocrit and MCV in the blood of professional radiation workers were assessed and compared with that of non-radiation workers as a biological dosimetry.

All data were analyzed using software SPSS for Windows, version 20 to assess the group statistics for radiation workers and controls such as mean percent and mean±SD. Comparison between groups was carried out by two-sided Student’s t-test. In all analyses, a significance level of 0.05 was adopted. 

## Results and Discussion

The exposed subjects include 11 Nuclear Medicine (27.5 percent), 24 Radiology (60 percent) and 5 Radiotherapy (12.5 percent) workers. The study group consisted of 14 males (35 percent) and 26 females (65 percent). The mean age of subjects was 34.5 ± 7.54 and 34.52 ± 7.99 in controls. Demographic characteristics of exposed workers are presented in Table 1.

**Table 1:** General Characteristics of Study Group

**Parameters**	**Exposed Subjects** **N (%)**	**Controls** **N (%)**
**Number of Individuals**	40	40
**Mean Age (Range)**	34.5±7.54	34.52±7.99
**Gender**		
Male	14 (35%)	26 (65%)
Female	14 (35%)	26 (65%)
**Exposed Category**		
Nuclear Medicine	11 (27.5%)	
Radiology	24 (60%)	
Radiotherapy	5 (12.5%)	
Duration of Employment*		
< 10 years	52 (65%)	
≥ 10 years	28 (35%)	
**Educational Certificate**		
Diploma	8 (20%)	
BSc & MSc	32 (80%)	
**Median Exposure**		
Nuclear Medicine	1.48±2.21	
Radiology	0.18±0.73	
Radiotherapy	0.21±0.35	

*Duration of Employment: Exposed and control subjects were matched with employment period.

The occupational record of radiation workers in diagnostic and therapeutic wards was at least two years and a maximum of 24 years with the mean of 9.02 ± 6.29. The occupational record in control group was 8.3 ± 5.55. Exposed subjects had a median exposure of 0.68±1.58 mSv/year by film badge dosimetry.

In this research, we found that there were not statistically significant differences in hematological parameters of professional radiation workers with less than 10 years records, as compared with controls (p>0.05) (Table 2).

**Table 2:** Comparison of mean blood parameters in two groups (case & control) with less than 10 years of occupational record (Group 1)

**Hematological Parameters**	**Group1**		**P-Value**
	**Case** **Mean ± SD**	**Control** **Mean ± SD**	
White Blood Cells	6.08 ± 1.42	6.51 ± 1.34	0.29
Lymphocytes	31.48 ± 7.31	34 ± 8.03	0.82
Monocytes	8.99 ± 1.38	8.39 ± 1.72	0.23
Neutrophils	53.31 ± 8.94	55 ± 9.09	0.52
Red Blood Cells	4.55 ± 0.48	4.57 ± 0.44	0.95
Hemoglobin	13.22 ± 1.48	13.44 ± 1.38	0.85
Hematocrit	38.47 ± 3.54	38.84 ± 3.01	0.95
Mean Corpuscular Volume	84.4 ± 4.55	85.11 ± 4.68	0.58
Platelets	239.6 ± 38.83	240.48 ± 41.31	0.94

Values significant at p<0.05

Statistically significant decreases in Hb and MCV were observed in professional radiation worker group with at least 10 years records as compared with controls (P<0.05) (Table 3).

**Table 3:** Comparison of mean blood parameters in two groups (case & control) with more than 10 years of occupational record (Group 2)

**Hematological Parameters**	**Group 2**		**P-Value**
	**Case** **Mean ± SD**	**Control** **Mean ± SD**	
White Blood Cells	6.53 0.93	6.97 1.6	0.35
Lymphocytes	36.02 7.66	35.23 5.35	0.68
Monocytes	7.66 1.37	8.05 1.62	0.52
Neutrophils	52.51 8.45	53.52 6.83	0.72
Red Blood Cells	5.04 0.39	4.91 0.41	0.455
Hemoglobin	13.82 1.56	14.36 1.47	0.008*
Hematocrit	40.38 3.70	41.17 3.46	0.14
Mean Corpuscular Volume	80.14 6.19	84 5.88	0.025*
Platelets	240.73 44.07	240 58.47	0.93

Values significant at p<0.05

The mean value of Red Blood Cells (RBCs) in personnel working in radiology departments seemed to show decrease in comparison with other radiation workers.

The effective annual dose ranged from 0.05 to 6.84 mSv; radiation workers had a median exposure of 0.68±1.58 mSv/year. These doses, although below maximal permissible limits set by the International Commission of Radiation Protection (ICRP), can have clear biological effects as suggested here by the decreased levels of Hb and MCV. Some studies showed decreased MCV in response to low-dose radiation while others showed increased MCV. This controversy may be related to the radiation dose or dose rate or blood parameters[1].

The data clearly show that irradiated Hb is significantly less than non-irradiated controls. Experiments conducted on Hb showed a similar decrease[1]. Statistically significant decrease or increase was not observed in the number of monocytes and neutrophils; this is probably because the absorbed dose was too low to affect phagocytes. Peripheral blood phagocytes have been considered to be relatively resistant to irradiation[14]. Statistically significant changes were not observed in the number of lymphocytes. These findings confirm the results of similar studies[15, 16]. The present study has shown no significant correlation between the number of platelets and exposure to chronic low doses of ionizing radiation. Studies of exposure to low-dose ionizing radiation have demonstrated the same results[17]. This phenomenon is known as radiation adaptive response[18]. Adaptive responses induced by low dose of radiation have been observed in hematopoietic and immune systems as shown by stimulatory effects on resistance to radiation-induced cytogenetic damage and cell growth[19]. The present study suggests that there are some changes in hematological parameters due to occupational exposure to chronic low doses of ionizing radiation in radiotherapy, radiology and nuclear medicine departments. Therefore, the exposed medical personnel should carefully follow the radiation protection instructions and try to minimize radiation exposure as much as possible.

Using dietary supplements containing antioxidant vitamins such as beta-carotene, vitamin C and E is recommended for protecting immune responses of radiation workers[20]. Antioxidants can also protect cell membrane from phagocytes such as neutrophils from oxidative effects of antioxidant compounds produced by radiation[20].

In occupational radiation accidents, in the presence and/or absence of physical dosimetry, the estimation of dose received by the exposed population in the work environment using the changes of hematological parameters, can be performed in the days following exposure in order to optimize the care of exposed individuals and to assess their health according to the received radiation dose. 

## Conclusion

Considering and following up the health of persons who are occupationally exposed to long-term low levels of ionizing radiation are of great importance. Therefore, the current study was conducted to investigate the biological effects of occupational exposure based on hematological parameters because according to the results biological dosimetry, based on the analysis of hematological parameters can be used as a supplement method for biological dosimetry based on chromosome aberrations (CA) and physical dosimetry, respectively.

In radiobiology, the hematopoietic system of the human body is concerned to be the most sensitive biological indicator; therefore, much more precise and careful studies concerning and considering different parameters should be carried out. 

## References

[1] Abdolmaleki A, Sanginabadi F, Rajabi A, Saberi R (2012). The Effect of Electromagnetic Waves Exposure on Blood Parameters. *International Journal of Hematology-Oncology and Stem Cell Research*.

[2] Erdal N, Gurgul S, Celik A (2007). Cytogenetic effects of extremely low frequency magnetic field on Wistar rat bone marrow. *Mutat Res*.

[3] Banik S, Bandyopadhyay S, Ganguly S (2003). Bioeffects of microwave--a brief review. *Bioresour Technol*.

[4] Calabrese EJ, Dhawan G, Kapoor R (2014). Use of X-rays to treat shoulder tendonitis/bursitis: a historical assessment. *Arch Toxicol*.

[5] Persson L (2000). *Effects of low--dose ionizing radiation*.

[6] Bashore T (2001). Fundamentals of X-ray imaging and radiation safety. *Catheter Cardiovasc Interv*.

[7] Feinendegen LE, Brooks AL, Morgan WF (2011). Biological consequences and health risks of low-level exposure to ionizing radiation: commentary on the workshop. *Health Phys*.

[8] Kopjar N, Garaj-Vrhovac V (2005). Assessment of DNA damage in nuclear medicine personnel--comparative study with the alkaline comet assay and the chromosome aberration test. *Int J Hyg Environ Health*.

[9] Ward E, Hornung R, Morris J, Rinsky R, Wild D, Halperin W ( 1996
). Risk of low red or white blood cell count related to estimated benzene exposure in a rubberworker cohort (1940-1975). *Am J Ind Med
*.

[10] Ropolo M, Balia C, Roggieri P, Lodi V, Nucci MC, Violante FS (2012). The micronucleus assay as a biological dosimeter in hospital workers exposed to low doses of ionizing radiation. *Mutat Res*.

[11] Torkabadi E, Kariminia A, Zakeri F (2007). Alteration of peripheral blood T-reg cells and cytokines production in angiography personnel exposed to scattered X-rays. *Iran J Allergy Asthma Immunol*.

[12] Rozgaj R, Kasuba V, Sentija K, Prlic I (1999). Radiation-induced chromosomal aberrations and haematological alterations in hospital workers. *Occup Med* (*Lond*).

[13] Shahid S, Mahmood N, Nawaz M, Sheikh S, Ahmad N (2014). Assessment of impacts of hematological parameters of chronic ionizing radiation exposed workers in hospitals. *FUUAST J Biol*.

[14] Buescher ES, Gallin JI (1984). Radiation effects on cultured human monocytes and on monocyte-derived macrophages. *Blood*.

[15] Rees GS, Daniel CP, Morris SD, Whitehouse CA, Binks K, MacGregor DH (2004). Occupational exposure to ionizing radiation has no effect on T- and B-cell total counts or percentages of helper, cytotoxic and activated T-cell subsets in the peripheral circulation of male radiation workers. *Int J Radiat Biol*.

[16] Schubauer-Berigan MK, Wenzl TB (2001). Leukemia mortality among radiation-exposed workers. *Occup Med*.

[17] Sayed D, Abd Elwanis ME, Abd Elhameed SY, Galal H (2011). Does occupational exposure to low-dose ionizing radiation affect bone marrow thrombopoiesis?. *Int Arch Med*.

[18] Kadhim MA, Moore SR, Goodwin EH (2004). Interrelationships amongst radiation-induced genomic instability, bystander effects, and the adaptive response. *Mutat Res*.

[19] Liu G, Gong P, Zhao H, Wang Z, Gong S, Cai L (2006). Effect of low-level radiation on the death of male germ cells. *Radiat Res*.

[20] Guan J, Stewart J, Ware JH, Zhou Z, Donahue JJ, Kennedy AR (2006). Effects of dietary supplements on the space radiation-induced reduction in total antioxidant status in CBA mice. *Radiat Res*.

[21] Klucinski P, Mazur B, Sedek L, Aptekorz M, Cieslik P, Hrycek AH (2014). Assessment of selected B cells populations in the workers of X-ray departments. *Int J Occup Med Environ Health*.

[22] Vral A, Fenech M, Thierens H (2011). The micronucleus assay as a biological dosimeter of in vivo ionising radiation exposure. *Mutagenesis*.

[23] Amaral A (
2005
). Physical and Biological Dosimetry for Risk Perception in Radioprotection. *Braz Arch Biol Technol*.

